# Combination Treatment of CI-994 With Etoposide Potentiates Anticancer Effects Through a Topoisomerase II-Dependent Mechanism in Atypical Teratoid/Rhabdoid Tumor (AT/RT)

**DOI:** 10.3389/fonc.2021.648023

**Published:** 2021-07-21

**Authors:** Hee Yeon Kim, Seung Ah Choi, Eun Jung Koh, Kyung Hyun Kim, Ji Hoon Phi, Ji Yeoun Lee, Seung-Ki Kim

**Affiliations:** ^1^ Division of Pediatric Neurosurgery, Pediatric Clinical Neuroscience Center, Seoul National University Children’s Hospital, Seoul, South Korea; ^2^ Department of Neurosurgery, Seoul National University Hospital, Seoul National University College of Medicine, Seoul, South Korea; ^3^ Department of Anatomy, Seoul National University College of Medicine, Seoul, South Korea

**Keywords:** atypical teratoid/rhabdoid tumor, combination treatment, HDAC1 inhibition, synergism, topoisomerase II

## Abstract

**Purpose:**

Atypical teratoid/rhabdoid tumor (AT/RT) is arising typically in young children and is associated with a dismal prognosis which there is currently no curative chemotherapeutic regimen. Based on previous studies showing high histone deacetylase 1 (HDAC1) expression in AT/RT, the HDAC1 inhibitor CI-994 was used as a novel treatment strategy in this study. We assessed the anticancer effects of CI-994 and conventional drugs (etoposide, cisplatin or 4-HC) in AT/RT cells.

**Methods:**

AT/RT patient-derived primary cultured cells and cell lines were prepared. HDAC1 was estimated by real-time quantitative polymerase chain reaction (RT-qPCR). The interaction of the drugs was analyzed using isobologram analysis. Cell viability, apoptosis, HDAC enzyme activity and western blot assays were carried out.

**Results:**

HDAC1 was overexpressed in AT/RT compared to medulloblastoma. The combination index (CI) of CI-994 with etoposide revealed a synergistic effect in all AT/RT cells, but no synergistic effect was observed between CI-994 and cisplatin or 4-HC. CI-994 effectively reduced not only Class I HDAC gene expression but also HDAC enzyme activity. The combination treatment of CI-994 with etoposide significantly increased apoptosis compared to the single treatment. The enhanced effect of apoptosis by this combination treatment is related to a signaling pathway which decreases topoisomerase (Topo) II and increases histone H3 acetylation (Ac-H3).

**Conclusion:**

We demonstrate that the combination treatment of CI-994 with etoposide exerts a synergistic anticancer effect against AT/RT by significantly inducing apoptosis through Topo II and Ac-H3 regulation.

**Clinical Relevance:**

This combination treatment might be considered a viable therapeutic strategy for AT/RT patients.

## Introduction

Atypical teratoid/rhabdoid tumor (AT/RT) is one of the most malignant pediatric brain tumors that typically arises in infants younger than 3 years old (1). Maximal safe resection followed by multimodal therapy is recommended as the standard treatment. However, the prognosis of patients with AT/RT is still poor (2, 3). The difficulty of gross total resection, incomplete efficacy of intensive chemotherapy and limitation of radiotherapy for young patients highlight the urgency of developing novel therapeutic strategies (3).

AT/RT is characterized by biallelic loss-of-function alterations in *SMARCB1*, which encodes the hSNF5/BAF47/INI1 subunit of the SWI/SNF chromatin remodeling complex (3, 4). Previous in-depth molecular studies explained the observed clinical heterogeneity but relatively unaltered genome of AT/RT by noting substantial heterogeneity in epigenetic profiles (2, 3). The Toronto group (5) and German group (6) recently classified AT/RT into 3 molecular subgroups based on its epigenetic profiles from two different perspectives. Studies targeting the mechanisms of epigenetic regulation in AT/RT treatment have been extensively conducted, leading to successful use of histone deacetylase (HDAC) inhibitors such as trichostatin A, SAHA, and SNDX-275 (7). It also suggested that a specific class of HDAC inhibitors may be more effective for certain molecular classes of AT/RT (5).

HDAC regulates the expression of genes and proteins involved in both cancer initiation and progression (8), and high expression levels of several HDACs are associated with poor prognosis of cancer patients (9). Additionally, HDACs have been found to regulate cancer cell functions, including DNA damage, cell death and differentiation (10). Therefore, the anticancer effects of HDAC inhibitors have been evaluated in various cancers (11).

HDAC1, which is a Class I HDAC, has been reported to play important roles in epigenetic regulation for tumor progression and is significantly overexpressed in many cancers (10, 12), including AT/RT (13, 14). Importantly, HDAC1 is highly expressed in AT/RT tissues compared to normal cerebellum and CNS non-cerebellum (15). As a drug that can inhibit HDAC1, CI-994 (Tacedinaline, N-acetyldinaline) is an oral compound that is also a selective Class I HDAC inhibitor (16). CI-994 has been verified to exhibit significant anticancer activity against a broad spectrum of human cancers *in vitro* (17) and *in vivo* (16).

Many preclinical and clinical studies have examined rational combinations of HDAC inhibitors with many current therapies for the treatment of hematological and solid tumor malignancies (18). Notably, CI-994 was investigated in combination with other anticancer drugs in phase I/II clinical trials for solid tumors (19). Therefore, the potential benefits that CI-994 might confer in the treatment of AT/RT led us to investigate the combination treatment of CI-994 with conventional anticancer drugs.

In this study, we evaluated the combination treatments of CI-994 with three different conventional chemotherapeutic agents (etoposide, cisplatin, or ifosfamide) commonly used in two protocols for the treatment of AT/RT (2). As etoposide showed the most potent synergistic effect with CI-994, we investigated the potential signaling pathway affected by this combination that leads to its anticancer effects.

## Materials and Methods

### Patients and Samples

Brain tumor tissues (**Table 1**) were collected from patients diagnosed with AT/RT (N=13) and MBL (N=13) who underwent initial surgery at the Seoul National University Children’s Hospital. The Institutional Review Board (IRB) of the Seoul National University Hospital (SNUH) approved the study protocol (IRB approval No. 1707-095-878). The pathological diagnosis of AT/RT was made histologically and confirmed by the lack of INI-1/SMARCB1 protein expression. The AT/RT subgroup was determined by immunohistochemistry staining of tissues (6). The molecular groups of medulloblastoma were analyzed by NanoString nCounter (20).

**Table 1:** Patient information.

Sample	Gender	Age	Location	M stage	Subtypes
**SNU.AT/RT-1**	M	13m	Lt. CPA	M3	MYC
**SNU.AT/RT-2**	M	18m	Vermis	M3	TYR/MYC
**SNU.AT/RT-3**	F	32m	Rt. CPA	M0	MYC
**SNU.AT/RT-4**	M	17m	Vermis	M0	SHH
**SNU.AT/RT-5**	M	20m	Lt. LV	M3	TYR/MYC
**SNU.AT/RT-6**	F	2	Lt. cbll	M0	TYR/MYC
**SNU.AT/RT-7**	F	2m	Rt. cbll	M0	TYR/MYC
**SNU.AT/RT-8**	M	11m	Lt. LV	M3	TYR/MYC
**SNU.AT/RT-9**	F	2m	Rt. cbll	M3	TYR/MYC
**SNU.AT/RT-10**	M	10m	4V	M0	TYR
**SNU.AT/RT-11**	M	28m	Rt. parietal	M0	undefined
**SNU.AT/RT-12**	F	14m	Rt. CPA	M3	undefined
**SNU.AT/RT-13**	M	23m	Rt. LV	M0	undefined
**SNU.MBL-1**	M	3	4V	M3	Group3
**SNU.MBL-2**	F	3	4V	M2	Group3
**SNU.MBL-3**	M	8	4V	M0	WNT
**SNU.MBL-4**	F	7	4V	M0	WNT
**SNU.MBL-5**	F	31m	4V	M3	Group4
**SNU.MBL-6**	M	7	4V	M0	Group4
**SNU.MBL-7**	M	7	4V	M1	Group4
**SNU.MBL-8**	F	17m	4V	M0	SHH
**SNU.MBL-9**	M	9m	Lt. cbll	M0	SHH
**SNU.MBL-10**	M	8	Rt. cbll	M0	SHH
**SNU.MBL-11**	F	4	4V	M0	undefined
**SNU.MBL-12**	F	15	Rt. CPA	M0	undefined
**SNU.MBL-13**	M	11	4V	M0	undefined

MB, medulloblastoma; AT/RT, atypical teratoid/rhabdoid tumor; M, male; F, female; m, month; Rt, right; Lt, left; V, ventricle; cbll, cerebellum; LV, lateral ventricle.

### Real-Time Quantitative Polymerase Chain Reaction (RT-qPCR)

Total RNA was isolated using the miRNeasy Mini Kit (Qiagen, Hilden, Germany), and cDNA was synthesized using the EcoDry Premix kit (Clontech, Mountain View, CA) (21). RT-qPCR assay was performed by a TaqMan assay on an ABI 7500 system (Applied Biosystems, Foster City, CA) using TaqMan probes for HDAC1, HDAC2, HDAC3, HDAC8, and GAPDH. The relative expression levels in each sample were calculated and quantified by using the 2^-ΔΔCT^ method. The value of each control sample was set to one and was used to calculate the fold change in target gene expression. GAPDH was utilized to normalize the gene expression results.

### Immunohistochemistry (IHC)

The expression of HDAC1 protein within tissues were verified by IHC as previously described (6). A total of 8 cases of tissue (4 cases in medulloblastoma and 4 cases in AT/RT) used to verify HDAC1 protein expression. Of these, 3 cases of each group were newly obtained, and 1 case of each group was included in the previous RT-qPCR analysis. Briefly, the sections were incubated with primary antibodies, HDAC1 (1:1000, Abcam, Cambridge, MA), for 32 min at 37°C, and a secondary antibody for 20 min at 37°C. The stained sections were detected using the Ventana ChromoMap Kit (Ventana Medical Systems) and discovered using XT automated IHC strainer (Ventana Medical Systems, Oro Valley, AZ).

### Cell Culture

AT/RT primary cells were cultured as previously described (21). The cell lines of AT/RT (BT12 and BT 16) and MBL (UW228 and MED8A) were provided from Dr. Peter Houghton (Nationwide Children’s Hospital) and Dr. Young Shin Ra (Asan Medical Center, Seoul, Korea), respectively. The human neural stem cell HB1.F3 was used as a normal control. All cells were cultured in Dulbecco’s modified Eagle’s medium (DMEM; Welgene, Seoul, Korea) containing 10% fetal bovine serum and 1% antibiotic-antimycotics and incubated at 37°C in a 5% CO_2_ atmosphere.

### Drugs

CI-994, etoposide, and cisplatin were purchased from Selleckchem (Houston, TX). We used the activated form of ifosfamide, 4-hydroperoxycyclophosphamide (4-HC), from Cayman (Ann Arbor, MI). The drugs were dissolved in dimethyl sulfoxide (DMSO) to generate 10 mM stock solutions and diluted to the indicated concentrations with culture medium before the experiments.

### Cell Viability Assays

The median inhibitory concentration (IC_50_) was determined in AT/RT cells. The cells (4 × 10^3^) were cultured in 96-well plates and exposed to various concentrations of the drugs (0-100 µM). Cells treated with 0.1% DMSO were used as a control. Cell viability was measured using the EZ-cytox kit (Daeil Lab Service, Seoul, Korea) after drug treatment for 72 h. The percentage of cell viability of the treated cells was measured relative to that of the control cells. Cell growth curves were drawn, and the IC_50_ was calculated by nonlinear regression analysis using Prism software (La Jolla, CA).

### Isobologram Analysis

To evaluate the dose-responses of the CI-994-based combination treatments, an isobologram was drawn for each drug combination based on 5 constant ratios: 0.25× IC_50_, 0.5× IC_50_, IC_50_, 2× IC_50_, and 4× IC_50_ (22). The synergy, additivity or antagonism was calculated on the basis of the multiple drug effect equation and quantified by the combination index (CI) and fraction affected (Fa) according to the Chou-Talalay algorithm utilizing CompuSyn software (Paramus, NJ, www.combosyn.com) (22, 23). The CI values indicate synergistic (CI < 1), additive (CI = 1) or antagonistic effects (CI > 1). The Fa levels of 50% (Fa = 0.5), 70% (Fa = 0.7), and 80% (Fa = 0.8) inhibition were created to study the dose-dependent interaction of the drug combinations. Fa < 0.5 was regarded as irrelevant because a large fraction of the cell population showed proliferation and reduced growth inhibition.

### HDAC Enzyme Activity Analysis

HDAC enzyme activity was assessed by an HDAC enzyme activity kit (Biovision, Mountain View, CA) (23). After 72 h of drug treatment, proteins (50 μg) extracted from the cells was mixed with the assay substrate and incubated at 37°C for 1 h. The reaction was stopped by adding 10 μl of lysine developer and incubated for an additional 30 min at 37°C. Test samples were measured by a fluorimeter (Molecular Devices, Sunnyvale, CA) at 405 nm.

### Apoptosis Analysis

Apoptosis was evaluated by the Annexin V-Fluorescein isothiocyanate (FITC)/propidium iodide (PI) binding assay kit (BD Biosciences, Franklin Lakes, NJ) according to the manufacturer’s instructions. After drug treatment for 48 h, the cells (1 × 10^6^ cells/ml) were harvested, stained with Annexin V and PI in the dark for 15 min, subjected to FACSCanto (BD), and analyzed by FlowJo software.

### Western Blot

Total proteins were extracted using radioimmunoprecipitation (RIPA) lysis buffer. Western blotting was performed using the iBlot system (Invitrogen) as previously described (23). The following primary antibodies were used: topoisomerase II (Topo II, 1:5000, Abcam), acetylated histone H3 (Ac-H3, 1:2000, Abcam), γ-H2AX (1:5000, Abcam), cleaved Parp (1:1000, Cell Signaling Technology, Danvers, MA), active Caspase-3 (cleaved Caspase-3, 1:100, Millipore, MA), Survivin (1:5000, Abcam), NF-κB (1:500, Abcam), C-Myc (1:10000, Abcam) and β-actin (1:5000, Sigma-Aldrich, St. Louis, MO). The blots were visualized by enhanced chemiluminescence (ECL, Invitrogen) with X-ray film. The band intensities were quantified using ImageJ software and normalized to β-actin.

### Statistical Analysis

All the values were calculated as the mean ± SD or expressed as the percentage ± SD of the controls. Multiple group comparisons were performed by 1-way ANOVA. Differences between 2 groups were determined using a 2-tailed Student’s *t*-test and Mann-Whitney test. GraphPad Prism v7.0 software was used for all the statistical analyses. All the analyses were repeated at least three times, and differences were considered statistically significant at p < 0.05.

## Results

### Overexpression of HDAC1 in AT/RT

**Figure 1:**
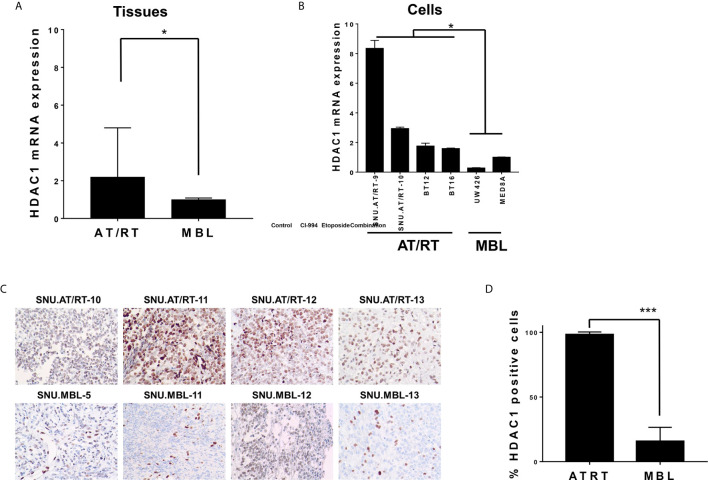
HDAC1 expression in AT/RT samples compared to MBL samples. **(A)** The quantitative polymerase chain reaction (qPCR) showed that HDAC1 mRNA expression in AT/RT tissues was 2.19 (p = 0.027) folds higher than in MB tissues. **(B)** HDAC1 mRNA level was tested in 3 AT/RT primary cultured cells(SNU.AT/RT-5, SNU.AT/RT-9, SNU.AT/RT-10) and each established AT/RT (BT12, BT16) and MB (UW426, MED8A) cell lines. HDAC1 is significantly overexpressed in AT/RT samples compared to the lowest expression level in UW426, one of MB cell lines. **(C)** IHC results show significantly higher HDAC1 protein expression in AT/RT compared to medulloblastoma. **(D)** The graph shows the percentage of HDAC1 positive cells in IHC (p < 0.0001). *p < 0.05, ***p < 0.0001.

We first evaluated the mRNA expression levels of HDAC1 in AT/RT tissues and cells. Compared with MBL tissues, AT/RT tissues exhibited increased HDAC1 mRNA expression (2.19-fold, p < 0.05, **Figure 1A**) and protein expression (**Figures 1C** **, D**). In addition, we confirmed that there was no significant change in HDAC1 mRNA expression depending on the MYC subgroup (*p*= NS, **Supplementary Figure S1**). HDAC1 was more highly expressed in all AT/RT cells than in MBL cells (6-fold p < 0.05, **Figure 1B**).

### Determination of IC_50_ Values

**Figure 2:**
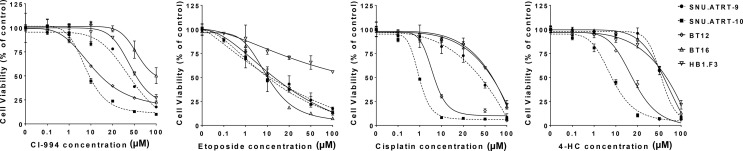
Single treatment in AT/RT cells. The viability of AT/RT cells against selected drugs (CI-994, etoposide, cisplatin, or 4-HC) was assessed by estimating their IC50.

The prerequisite for confirming a synergistic effect is to determine the potency of each drug and the slopes of their concentration response curves. Therefore, we investigated the IC_50_ values of each drug in primary cultured AT/RT cells (SNU.AT/RT-9 and SNU.AT/RT-10) and AT/RT cell lines (BT12 and BT16). Increasing concentrations of each drug significantly reduced the viability of all AT/RT cells in a dose-dependent manner (**Figure 2**). The IC_50_ values ranged from 7.5 ± 0.2 to 65.0 ± 22.0 µM for CI-994, 4.9 ± 2.4 to 13.4 ± 4.3 µM for etoposide, 1.0 ± 0.05 to 56.1 ± 7.5 µM for cisplatin, and 5.3 ± 0.2 to 57.4 ± 5.0 µM for 4-HC in AT/RT cells (**Table 2**). The IC_50_ values of HB.F3 cells were 48.1 ± 26.6 µM for CI-994, 16.3 ± 5.2 µM for etoposide, 62.8 ± 3.8 µM for cisplatin, and 25.7 ± 11.1 µM for 4-HC. Compared to AT/RT cells, HB.F3 cells were more resistant to etoposide and cisplatin.

**Table 2:** IC_50_ of each drug in AT/RT cell lines.

Cell lines	CI-994	Etoposide	Cisplatin	4-HC*
**SNU.AT/RT-9**	40.4 ± 10.4µM	13.4 ± 4.3µM	25.1 ± 4.1µM	56.5 ± 16.8µM
**SNU.AT/RT-10**	7.5 ± 0.2µM	9.9 ± 0.4µM	1.0 ± 0.05µM	5.3 ± 0.2µM
**BT12**	36.1 ± 1.5µM	9.2 ± 0.7µM	4.7 ± 1.1µM	15.7 ± 0.3µM
**BT16**	65.0 ± 22.0µM	4.9 ± 2.4µM	56.1 ± 7.5µM	57.4 ± 5.0µM
HB1.F3**	48.1 ± 26.6µM	16.3 ± 5.2µM	62.8 ± 3.8µM	25.7 ± 11.1µM

*Activated form of ifosfamide, **Neural stem cells.

### Synergistic Effect of the Combination Treatment of CI-994 With Etoposide Against AT/RT

**Figure 3:**
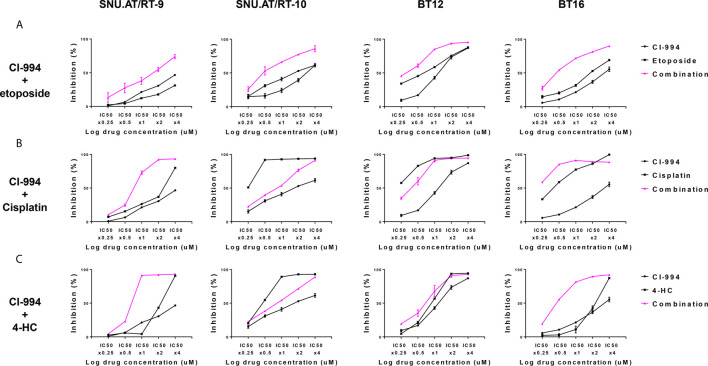
Comparative evaluation of the combination effect of CI-994 with conventional anticancer drugs. Curve shift analysis of CI-994 with conventional drugs (etoposide, cisplatin and 4-HC) in AT/RT cells shows that the degree of left shift represented the amount of synergism associated with the indicated drug combination. Single treatment: Black lines, Combination treatment: Pink lines. **(A)** In response to the combination treatment of CI-994 with etoposide, synergistic effects were observed and inhibited the growth potential of all AT/RT cells in a dose-dependent manner. **(B, C)** The combination treatment of CI-994 with cisplatin or 4 HC exerted cell type-specific effects. Synergistic effects were observed in the cells treated with the combination of CI-994 with cisplatin only for SNU.AT/RT-9 cells and in cells treated with the combination of CI-994 with 4-HC for SNU.AT/RT-9 cells and BT-16 cells.

To determine the drug interaction of CI-994 with conventional chemotherapeutic agents, we calculated the fraction affected (Fa) and combination index (CI) values. The effect of the combination treatment of CI-994 with etoposide in all AT/RT cells was interpreted as synergistic: 0.3-0.54 in SNU.AT/RT-9 cells, 0.09-0.13 in SNU.AT/RT-10 cells, 0.5-0.8 in BT12 cells and 0.32-0.49 in BT16 cells (**Figure 3A** and **Supplementary Table S1**). The combination treatment of CI-994 with cisplatin or 4-HC did not exert synergistic effects in some cells (**Figures 3B** **, C**). In particular, antagonism was observed in SNU.AT/RT-10 cells with the combination treatment of CI-994 with cisplatin (**Supplementary Table S2**) and in SNU.AT/RT-10 cells and BT12 cells with the combination treatment of CI-994 with 4-HC (**Supplementary Table S3**). Therefore, etoposide was chosen as the drug to be combined with CI-994.

**Figure 4:**
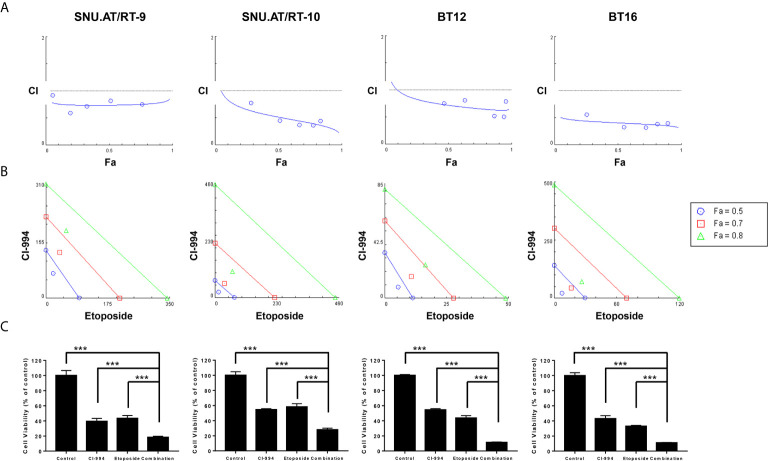
Synergistic effect of the combination treatment of CI-994 with etoposide in atypical teratoid/rhabdoid tumor (AT/RT) cells. Fraction affected *vs.* combination index (Fa-CI) plot was calculated for the combination of CI-994 with etoposide using an isobologram. **(A)** Dose-response curve shows that if the combination points (blue lines) lie below the gray line, there is a synergistic effect. Low CI values with increased Fa values suggest better compatibility and high synergism between CI-994 and etoposide. Graphs show a synergistic effect in all AT/RT cells. **(B)** The blue, red and green lines represent the theoretical additive line and the 0.5, 0.7 and 0.8 values, respectively. The synergy between CI-994 and etoposide increases as the calculated values approach the origin. In all AT/RT cells, synergism was observed at concentrations with Fa 0.5, Fa 0.7 and Fa 0.8 points. **(C)** Combination treatment of CI-994 with etoposide was significantly more effective than single treatment in all AT/RT cells. ***p < 0.0001.

At the optimal concentration of the combination treatment of CI-994 with etoposide, dose-response plots and Fa-CI were generated to the confirm drug interaction. The combination dose-response curves of all the AT/RT cells treated with CI-994 and etoposide shifted to the left, which indicated synergism by lowering the IC_50_ equivalent (**Figure 4A**). The Fa-CI plot showed all points to be under the horizontal line, which equaled 1 of the CI value at all concentrations. In the case of BT16 cells, all the points were closer to the bottom line than the points of the other AT/RT cells. The data points below the line of additivity indicate synergism (**Figure 4B**). The viability of cells exposed to the combination treatment of CI-994 with etoposide was significantly reduced by approximately 1.95- to 4.8-fold compared to that of cells exposed to the single treatment in all AT/RT cells (p < 0.0001, **Figure 4C**).

### Inhibition of HDAC1 mRNA Expression by CI-994

**Figure 5:**
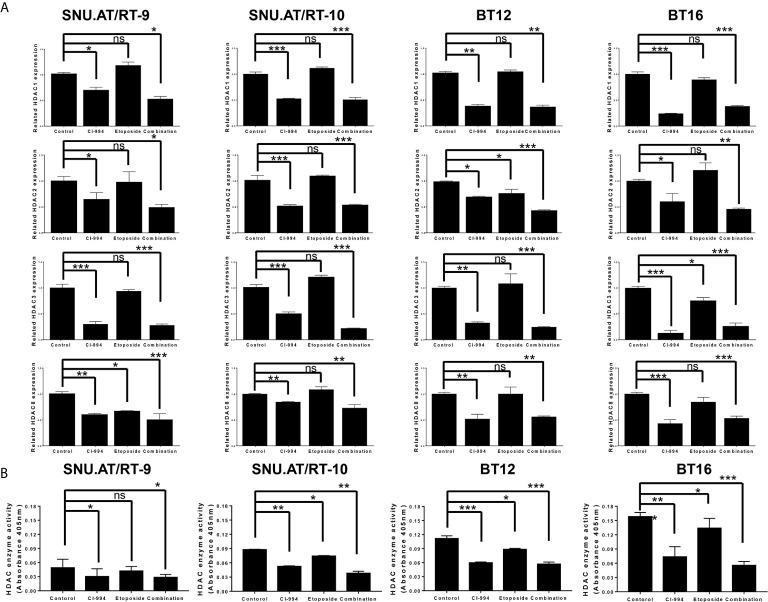
Class I histone deacetylase (HDAC) mRNA expression and HDAC enzyme activity following combination treatment of CI-994 with etoposide. **(A)** Class I HDAC expression was measured by RT-qPCR in AT/RT cells. Class I HDAC mRNA expression was significantly reduced by CI-994 treatment in all AT/RT cells. HDAC1 mRNA expression was not affected by etoposide single treatment in all AT/RT cells, but HDAC 2, 3, and 8 expression tended to decrease in some cells. **(B)** HDAC enzyme activities were significantly reduced by the CI-994 treatment and combination treatment. Even etoposide single treatment led to a slight decrease in SNU.AT/RT-10 cells, BT12 cells, and BT16 cells, but not in SNU.AT/RT-9. *p < 0.05, **p < 0.001, ***p < 0.0001, ns, not significant.

To confirm whether CI-994 effectively inhibits the mRNA expression of Class I HDAC, we performed RT-qPCR. As expected, HDAC1 expression was significantly decreased by CI-994 treatment alone and by the combination treatment compared to the control treatment in all AT/RT cells (**Figure 5A** and **Supplementary Table S4**). Etoposide single treatment did not affect HDAC1 mRNA expression. We also examined the mRNA expression of HDAC2, HDAC3 and HDAC8, which was significantly decreased by the CI-994 single treatment and combination treatment. In some AT/RT cells, HDAC2, HDAC3 or HDAC8 expression was reduced by etoposide single treatment.

### Decreased Activity of HDAC Following Combination Treatment of CI-994 With Etoposide

The inhibitory effect of the combination treatment of CI-994 with etoposide on HDAC activity was evaluated. Compared to the control treatment, the CI-994 single treatment and combination treatment effectively decreased the enzyme activities in all AT/RT cells (**Figure 5B** and **Supplementary Table S5**). Interestingly, the etoposide single treatment slightly suppressed HDAC enzyme activity in SNU.AT/RT-10 cells, BT12 cells, and BT16 cells, but not in SNU.AT/RT-9 cells.

### Enhanced Apoptosis Following Combination Treatment With CI-994 and Etoposide

**Figure 6:**
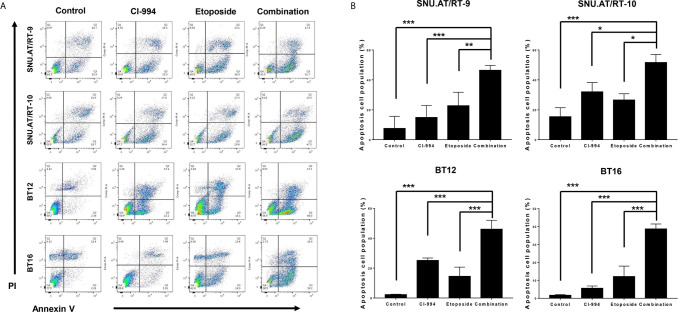
Effect of the combination treatment of CI-994 with etoposide on apoptosis in AT/RT cells. **(A)** Apoptosis in AT/RT cells was analyzed by Annexin V-FITC/propidium iodide assay and flow cytometry. **(B)** Percentages of apoptotic cells are presented on graphs. Combination of CI-994 with etoposide more considerably induced apoptosis in all AT/RT cells than the single treatment. *p < 0.05, **p < 0.001, ***p < 0.0001.

To confirm whether the synergistic anticancer effect of the combination treatment of CI-994 with etoposide is associated with apoptosis, we analyzed the proportion of apoptotic cells by Annexin V-FITC/PI binding assay. The percentage of apoptotic cells was significantly increased in all AT/RT cells treated with CI-994, etoposide and the combination. Importantly, the combination treatment induced more early apoptosis than CI-994 or etoposide single treatment in all AT/RT cells (**Figures 6A, B** and **Supplementary Table S6**).

### Decreased Topoisomerase II Expression and Increased Histone H3 Acetylation Following Combination Treatment With CI-994 and Etoposide

**Figure 7:**
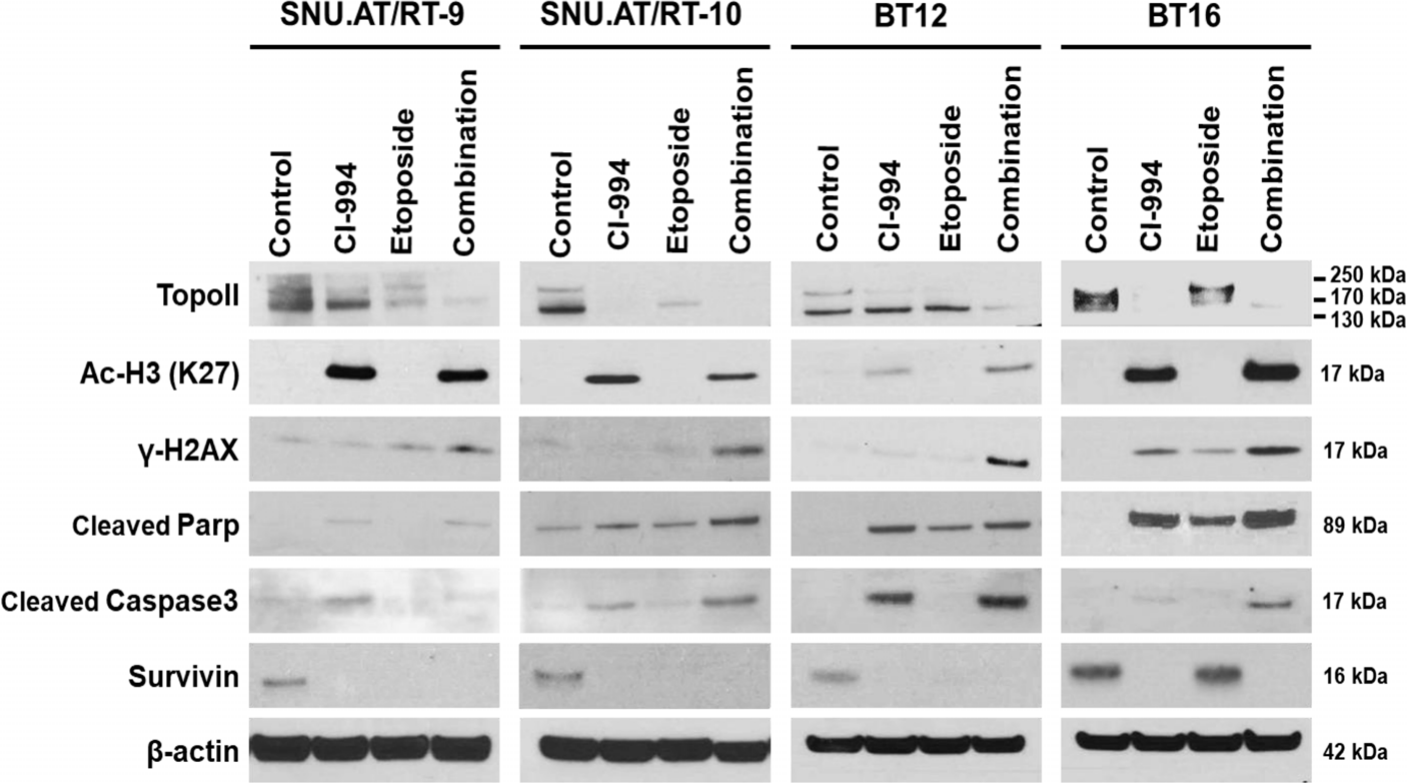
Protein expression involved in the synergistic effect of the combination treatment of CI-994 with etoposide. Western blot analysis shows the levels of topoisomerase (Topo) II, histone 3 acetylation (Ac-H3), γ-H2AX, cleaved Parp, cleaved Caspase-3 and Survivin in response to the combination treatment of CI-994 with etoposide.

**Figure 8:**
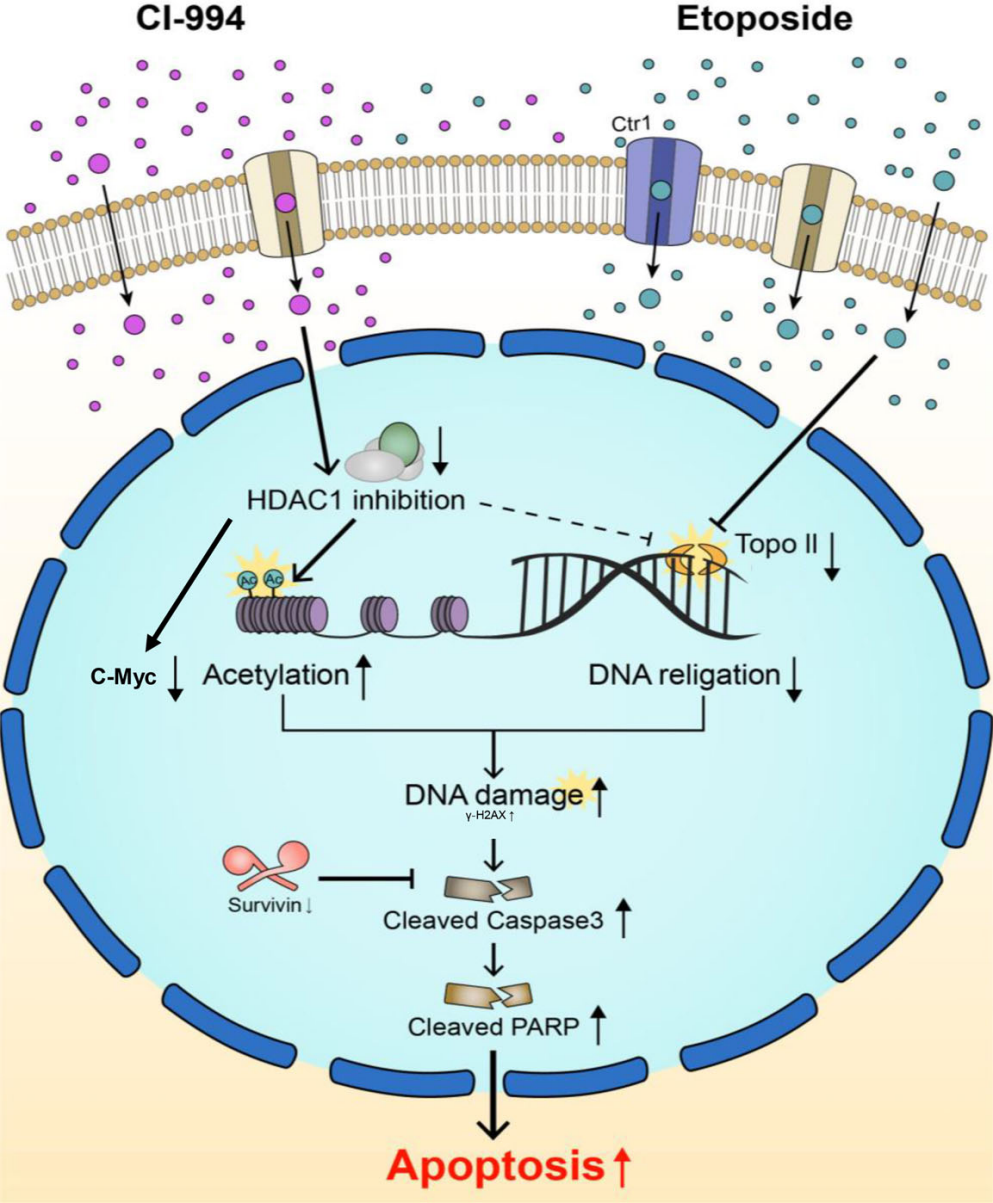
Schematic diagram of two main targets of the combination treatment. Topo II and HDAC1 are known to interact with each other and modify each other’s activity. CI-994 and etoposide can pass through cell membranes using transporters and/or passive diffusion. CI-994 enters the cell, inhibits HDAC1 enzyme activity and increases histone acetylation, therefore activating cleaved Parp. CI-994 also reduced C-Myc, which was more effective in combination treatment with etoposide. CI-994 can also increase γ–H2AX expression. Etoposide increases γ-H2AX expression, which not only increases cleaved Parp levels but also decreases Survivin levels. The action of each of these drugs through the regulation of Topo II and Ac-H3 synergistically increases cleaved Parp levels and cleaved Caspase-3 levels, leading to the apoptosis of AT/RT cells.

To investigate the molecular mechanisms associated with the synergistic anticancer effect of the combination treatment of CI-994 with etoposide on AT/RT cells, we explored the signaling pathways associated with DNA damage induced by Topo II and H3 acetylation (Ac-H3). In the majority of AT/RT cells, compared with the control and single treatments, the combination treatment led to decreased protein expression of Topo II and increased expression of Ac-H3, γ-H2AX, cleaved Parp, and cleaved Caspase-3 (**Figure 7** and **Supplementary Figure S2**). Survivin was decreased in response to the single and combination treatment in most AT/RT cells; however, in BT16 cells, Survivin expression was increased in response to the etoposide single treatment. Since CI-994 increased Ac-H3 expression, Ac-H3 expression may induce the expression of the DNA damage-related protein γ-H2AX and initiation of early apoptosis, which increases the levels of cleaved Parp and Caspase-3. We also confirmed changes in the expression of NF-κB and C-Myc after drug treatment. The combination treatment of CI-994 and etoposide did not show any difference in the expression of NF-κB (**Supplementary Figure S3A**) but C-Myc (**Supplementary Figure S3B**) was effectively decreased in all AT/RT cells. On the other hand, the etoposide single treatment did not affect Ac-H3 expression but increased γ-H2AX expression. These data suggested that the combination treatment enhanced DNA breakdown by decreasing Topo II expression and increasing Ac-H3 expression, which potentiated apoptosis (**Figure 8**).

## Discussion

Our study demonstrated the synergistic anticancer effect of the combination treatment of CI-994 with etoposide on AT/RT. The underlying mechanism of action was thought to occur through enhanced apoptosis due to decreased expression of Topo II and increased expression of Ac-H3.

The importance of HDAC-targeted therapeutics in various tumors (8, 24) has led to the extended use of specific HDAC inhibitors in pediatric brain tumors, including AT/RT (14). Previous studies showing HDAC1 overexpression in AT/RT (13) and verification in our samples provided rationale for the use of HDAC1 inhibitors. Since there are no commercially available inhibitors that regulate only HDAC1, we used CI-994, which is a relatively selective HDAC1 inhibitor (25, 26). It would be difficult to target and selectively regulate only HDAC1 because HDAC1, 2, 3 and 8, which are Class I HDACs, interact with each other (27). This limitation highlights the importance of intensive research on the development of HDAC single isoform inhibitors. This is one of the limitations of our study and remains a challenge to be addressed.

The three molecular subgroups of AT/RT are well known, and this should be taken into account as different subgroups may induce different drug sensitivities (28). The subgroups of AT/RT cells we used in this study were different (SNU.AT/RT-9: TYR/MYC, SNU.AT/RT-10: TYR, BT12: TYR, BT16: controversial). It should be kept in mind that the sensitivity of the drug may vary depending on the subgroup.

The penetration of CI-994 through the blood-brain barrier (BBB) is low (permeability surface area products BBB: 12.7 ± 0.1 μL/min/g brain) (29). Therefore, delivery strategies *via* intratumoral (30), intracisternal (31) and intranasal (32) injections may be required to bypass the BBB.

Many preclinical studies have suggested that the combination treatment of HDAC inhibitors with conventional chemotherapeutics safely shows synergistic effects even at lower concentrations (18). Recent studies have suggested that the use of HDAC inhibitors may be beneficial for the treatment of children with AT/RT as part of multimodal therapies (14). Although CI-994 is a potential anticancer drug, it is associated with dose-dependent toxicity and side effects, including thrombocytopenia and neutropenia (19, 26). Therefore, we investigated the drug interactions between CI-994 and conventional anticancer drugs (etoposide, cisplatin and 4-HC) that are commonly used for the treatment of AT/RT patients (23). We used isobologram analysis to determine which anticancer drugs have synergistic effects when combined with CI-994. Among the three combinations, the combination treatment of CI-994 with etoposide exerted the strongest effect in a dose-dependent manner. Despite the molecular heterogeneity of AT/RT tumors, isobologram analysis of the combination treatment of CI-994 with etoposide showed consistent responses in all AT/RT cells. On the other hand, the combination of CI-994 with cisplatin or 4-HC revealed antagonism in at least one combination ratio in AT/RT cells, and the response of each cell was inconsistent. For these reasons, we conducted this study focusing on the combination treatment of CI-994 with etoposide.

Next, we determined whether CI-994 efficiently inhibits Class I HDAC gene expression and enzyme activities in AT/RT cells. The inhibitory effect of the combination treatment of CI-994 with etoposide was relatively similar to the effect of CI-994 alone. As a result, there might be other regulatory factors that could cause the synergistic effect of the combination treatment. We speculated that two target molecules modify each other’s activity, thus generating the observed synergy. Previous studies have suggested that acetylation seems to influence the efficacy of etoposide, as both HDAC and histone acetyltransferase (HAT) activities increase the efficacy of etoposide (33). There is further evidence that HDAC1/2 complexes with Topo II modify each other’s activity *in vitro* and *in vivo* (34). Topo II-associated HDAC activity was reduced by a specific HDAC inhibitor, and the combination treatment could increase cytotoxicity (34). Etoposide induces apoptosis by inhibiting the Topo II cleavage complexes, leading to the accumulation of DNA damage (33). In addition, CI-994 and other HDACi have shown to induce apoptosis through post-induction suppression of NF-κB or C-Myc-mediated transcription (35, 36). Taken together, we assumed that the synergistic interaction between CI-994 and etoposide is mediated through Topo II (37). Our results showed considerably decreased Topo II expression and increased Ac-H3 expression in response to the combination treatment, suggesting the possibility that apoptosis may be elevated by these signaling pathways. C-Myc may be involved in this signal pathways, not by NF-κB related signal pathway.

In summary, our results demonstrate that the combination treatment of CI-994 with etoposide exerts a synergistic anticancer effect by promoting apoptosis of AT/RT cells through the modulation of Topo II and Ac-H3 *in vitro*. Although the use of more selective HDAC1 inhibitors and the addition of animal experiments remain challenges, our results support the possibility that the combination treatment of CI-994 with etoposide deserves further attention as a therapeutic option for pediatric AT/RT.

## Data Availability Statement

The original contributions presented in the study are included in the article/**Supplementary Material**. Further inquiries can be directed to the corresponding author.

## Ethics Statement

The studies involving human samples were reviewed and approved by the Institutional Review Board (IRB) of the Seoul National University Hospital (SNUH IRB approval No. 1707-095-878). The patients/participants provided their written informed consent to participate in this study.

## Author Contributions

S-KK supervised this study. SC designed the experiment. HK performed the experiment. SC and EK analyzed the data. SC and HK wrote the manuscript. JP, K-HK, SC and JL reviewed and edited the draft. All authors contributed to the article and approved the submitted version.

## Funding

This work was supported by the National Research Foundation of Korea (NRF) grant funded by the Korean government (MSIT) (2019R1F1A1063068) and by grants from the National Cancer Center, Republic of Korea (NCC-1810861-1).

## Conflict of Interest

The authors declare that the research was conducted in the absence of any commercial or financial relationships that could be construed as a potential conflict of interest.
