# Supplementary material for: Combination Treatment of CI-994 With Etoposide Potentiates Anticancer Effects Through a Topoisomerase II-Dependent Mechanism in Atypical Teratoid/Rhabdoid Tumor (AT/RT)
Source: Front Oncol. 2021 Jul 21;11:648023. doi: 10.3389/fonc.2021.648023 (PMC8337050; doi:10.3389/fonc.2021.648023)
Supplement: Supplementary file 1 [file DataSheet_1.docx]

**Supplementary Table S1. Calculation of drug interaction between CI-994 and etoposide by combination index (CI)**

| **Cell line** | **CI-994** | **Etoposide** | **Total Dose** | **Fa value** | **CI value** | **Interpretation** |
| --- | --- | --- | --- | --- | --- | --- |
|  | 10.1 µM | 3.3 µM | 13.4 µM | 0.2±0.004 | 0.30±0.005 | synergism |
|  | 20.2 µM | 6.7 µM | 26.9 µM | 0.3±0.004 | 0.43±0.005 | synergism |
| **SNU.AT/RT-9** | 40.4 µM | 13.4 µM | 53.8 µM | 0.5±0.004 | 0.52±0.008 | synergism |
|  | 80.8 µM | 26.8 µM | 107.6 µM | 0.7±0.004 | 0.51±0.019 | synergism |
|  | 161.6 µM | 53.5 µM | 215.1 µM | 0.9±0.004 | 0.54±0.015 | synergism |
|  | 1.9 µM | 2.5 µM | 4.3 µM | 0.3±0.023 | 0.12±0.015 | synergism |
|  | 3.7 µM | 4.9 µM | 8.7 µM | 0.5±0.018 | 0.11±0.010 | synergism |
| **SNU.AT/RT-10** | 7.5 µM | 9.9 µM | 17.3 µM | 0.6±0.014 | 0.13±0.009 | synergism |
|  | 14.9 µM | 19.7 µM | 34.7 µM | 0.7±0.013 | 0.12±0.010 | synergism |
|  | 29.9 µM | 39.4 µM | 69.3 µM | 0.9±0.015 | 0.09±0.014 | synergism |
|  | 9.0 µM | 2.3 µM | 11.3 µM | 0.5±0.009 | 0.54±0.014 | synergism |
|  | 18.1 µM | 4.6 µM | 22.6 µM | 0.6±0.020 | 0.66±0.062 | synergism |
| **BT12** | 36.1 µM | 9.2 µM | 45.3 µM | 0.9±0.011 | 0.50±0.066 | synergism |
|  | 72.2 µM | 18.4 µM | 90.5 µM | 0.9±0.003 | 0.51±0.087 | synergism |
|  | 144.4 µM | 36.8 µM | 181.0 µM | 1.0±0.000 | 0.80±0.160 | synergism |
|  | 16.3 µM | 1.2 µM | 17.5 µM | 0.3±0.012 | 0.41±0.023 | synergism |
|  | 32.5 µM | 2.4 µM | 34.9 µM | 0.5±0.004 | 0.33±0.005 | synergism |
| **BT16** | 65.0 µM | 4.9 µM | 69.9 µM | 0.7±0.008 | 0.32±0.010 | synergism |
|  | 130.0 µM | 9.8 µM | 139.8 µM | 0.8±0.007 | 0.37±0.014 | synergism |
|  | 260.0 µM | 19.6 µM | 279.5 µM | 0.9±0.004 | 0.49±0.014 | synergism |
