# Supplementary material for: Combination Treatment of CI-994 With Etoposide Potentiates Anticancer Effects Through a Topoisomerase II-Dependent Mechanism in Atypical Teratoid/Rhabdoid Tumor (AT/RT)
Source: Front Oncol. 2021 Jul 21;11:648023. doi: 10.3389/fonc.2021.648023 (PMC8337050; doi:10.3389/fonc.2021.648023)
Supplement: Supplementary file 2 [file DataSheet_2.docx]

**Supplementary Table S2. Calculation of drug interaction between CI-994 and cisplatin by combination index (CI)**

| **Cell line** | **CI-994** | **Cisplatin** | **Total Dose** | **Fa value** | **CI value** | **Interpretation** |
| --- | --- | --- | --- | --- | --- | --- |
|  | 10.1 µM | 6.3 µM | 16.4 µM | 0.1±0.006 | 19.14±2.264 | antagonism |
|  | 20.2 µM | 12.6 µM | 32.8 µM | 0.3±0.013 | 5.67±0.656 | antagonism |
| **SNU.AT/RT-9** | 40.4 µM | 25.1 µM | 65.5 µM | 0.7±0.020 | 0.41±0.056 | synergism |
|  | 80.8 µM | 50.2 µM | 131.0 µM | 0.9±0.002 | 0.11±0.005 | synergism |
|  | 161.6 µM | 100.4 µM | 262.0 µM | 0.9±0.001 | 0.17±0.002 | synergism |
|  | 1.9 µM | 0.25 µM | 2.1 µM | 0.3±0.002 | 2.34±0.035 | antagonism |
|  | 3.7 µM | 0.5 µM | 4.2 µM | 0.4±0.006 | 1.69±0.059 | antagonism |
| **SNU.AT/RT-10** | 7.5 µM | 1 µM | 8.5 µM | 0.5±0.006 | 1.66±0.043 | antagonism |
|  | 14.9 µM | 2 µM | 16.9 µM | 0.8±0.014 | 0.93±0.084 | synergism |
|  | 29.9 µM | 4 µM | 33.9 µM | 0.9±0.004 | 0.47±0.026 | synergism |
|  | 9.0 µM | 1.2 µM | 10.2 µM | 0.4±0.013 | 0.99±0.044 | synergism |
|  | 18.1 µM | 2.3 µM | 20.4 µM | 0.6±0.043 | 0.89±0.129 | synergism |
| **BT12** | 36.1 µM | 4.7 µM | 40.8 µM | 0.9±0.019 | 0.39±0.073 | synergism |
|  | 72.2 µM | 9.4 µM | 81.5 µM | 0.9±0.005 | 0.56±0.036 | synergism |
|  | 144.4 µM | 18.8 µM | 163.0 µM | 0.9±0.004 | 1.06±0.070 | antagonism |
|  | 16.3 µM | 14 µM | 30.3 µM | 0.6±0.004 | 0.41±0.005 | synergism |
|  | 32.5 µM | 28 µM | 60.5 µM | 0.9±0.004 | 0.26±0.004 | synergism |
| **BT16** | 65.0 µM | 56.1 µM | 121.1 µM | 0.9±0.001 | 0.32±0.002 | synergism |
|  | 130.0 µM | 112.1 µM | 242.1 µM | 0.9±0.002 | 0.78±0.015 | synergism |
|  | 260.0 µM | 224.3 µM | 484.2 µM | 0.9±0.002 | 1.63±0.026 | antagonism |
