# Supplementary material for: Combination Treatment of CI-994 With Etoposide Potentiates Anticancer Effects Through a Topoisomerase II-Dependent Mechanism in Atypical Teratoid/Rhabdoid Tumor (AT/RT)
Source: Front Oncol. 2021 Jul 21;11:648023. doi: 10.3389/fonc.2021.648023 (PMC8337050; doi:10.3389/fonc.2021.648023)
Supplement: Supplementary file 3 [file DataSheet_3.docx]

**Supplementary Table S3. Calculation of drug interaction between CI-994 and 4-HC by combination index (CI)**

| **Cell line** | **CI-994** | **4-HC** | **Total Dose** | **Fa value** | **CI value** | **Interpretation** |
| --- | --- | --- | --- | --- | --- | --- |
|  | 10.1 µM | 14 µM | 24.1 µM | 0.04±0.001 | 107.64±5.070 | antagonism |
|  | 20.2 µM | 28 µM | 48.2 µM | 0.2±0.009 | 5.55±0.512 | antagonism |
| **SNU.AT/RT-9** | 40.4 µM | 56 µM | 96.4 µM | 0.9±0.000 | 0.08±0.001 | synergism |
|  | 80.8 µM | 112 µM | 192.8 µM | 0.9±0.002 | 0.13±0.005 | synergism |
|  | 161.6 µM | 264.4 µM | 385.6 µM | 0.9±0.002 | 0.22±0.006 | synergism |
|  | 1.9 µM | 1.3 µM | 3.2 µM | 0.2±0.006 | 2.80±0.109 | antagonism |
|  | 3.7 µM | 2.6 µM | 6.4 µM | 0.4±0.05 | 2.30±0.055 | antagonism |
| **SNU.AT/RT-10** | 7.5 µM | 5.3 µM | 12.7 µM | 0.5±0.007 | 2.13±0.072 | antagonism |
|  | 14.9 µM | 10.5 µM | 25.5 µM | 0.7±0.010 | 1.75±0.099 | antagonism |
|  | 29.9 µM | 21.1 µM | 50.9 µM | 0.9±0.004 | 0.90±0.042 | synergism |
|  | 9.0 µM | 3.9 µM | 12.9 µM | 0.2±0.004 | 2.83±0.071 | antagonism |
|  | 18.1 µM | 7.8 µM | 25.9 µM | 0.4±0.039 | 2.85±0.463 | antagonism |
| **BT12** | 36.1 µM | 15.7 µM | 51.8 µM | 0.7±0.068 | 1.41±0.435 | antagonism |
|  | 72.2 µM | 31.4 µM | 103.5 µM | 0.9±0.022 | 0.64±0.156 | synergism |
|  | 144.4 µM | 62.8 µM | 207.1 µM | 0.9±0.008 | 0.99±0.107 | synergism |
|  | 16.3 µM | 14.4 µM | 30.6 µM | 0.2±0.009 | 1.67±0.080 | antagonism |
|  | 32.5 µM | 28.7 µM | 61.2 µM | 0.6±0.007 | 0.89±0.021 | synergism |
| **BT16** | 65.0 µM | 57.4 µM | 122.4 µM | 0.8±0.005 | 0.64±0.017 | synergism |
|  | 130.0 µM | 114.9 µM | 244.9 µM | 0.9±0.002 | 0.74±0.011 | synergism |
|  | 260.0 µM | 229.8 µM | 489.7 µM | 0.9±0.001 | 1.17±0.012 | antagonism |
