# Supplementary material for: Combination Treatment of CI-994 With Etoposide Potentiates Anticancer Effects Through a Topoisomerase II-Dependent Mechanism in Atypical Teratoid/Rhabdoid Tumor (AT/RT)
Source: Front Oncol. 2021 Jul 21;11:648023. doi: 10.3389/fonc.2021.648023 (PMC8337050; doi:10.3389/fonc.2021.648023)
Supplement: Supplementary file 4 [file DataSheet_4.docx]

**Supplementary Table S4. Comparison of HDAC class1 mRNA expression**

|  | **Cell line** | **Control**  **vs**  **Combination** | | **Control**  **vs**  **CI-994** | | **Control**  **vs**  **Etoposide** | |
| --- | --- | --- | --- | --- | --- | --- | --- |
| **HDAC1** | **SNU.AT/RT-9** | 1.79-fold | P = 0.0002 | 1.40-fold | P = 0.005 | 0.89-fold | P = 0.1369 |
|  | **SNU.AT/RT-10** | 1.98-fold | P <0.0001 | 1.90-fold | P <0.0001 | 0.90-fold | P = 0.0272 |
|  | **BT12** | 2.64-fold | P <0.0001 | 2.43-fold | P <0.0001 | 0.99-fold | P = 0.9993 |
|  | **BT16** | 2.63-fold | P <0.0001 | 4.18-fold | P <0.0001 | 1.12-fold | P = 0.0135 |
| **HDAC2** | **SNU.AT/RT-9** | 2.03-fold | P = 0.0023 | 1.63-fold | P = 0.0103 | 1.10-fold | P = 0.7090 |
|  | **SNU.AT/RT-10** | 1.88-fold | P < 0.0001 | 1.91-fold | P < 0.0001 | 0.92-fold | P = 0.1634 |
|  | **BT12** | 2.31-fold | P = 0.0001 | 1.42-fold | P = 0.0055 | 1.33-fold | P = 0.0108 |
|  | **BT16** | 2.18-fold | P = 0.0052 | 1.66-fold | P = 0.0151 | 0.83-fold | P = 0.2076 |
| **HDAC3** | **SNU.AT/RT-9** | 3.51-fold | P < 0.0001 | 3.43-fold | P < 0.0001 | 1.05-fold | P = 0.4057 |
|  | **SNU.AT/RT-10** | 4.56-fold | P < 0.0001 | 2.00-fold | P < 0.0001 | 0.83-fold | P = 0.0003 |
|  | **BT12** | 4.23-fold | P = 0.0021 | 3.15-fold | P = 0.0032 | 0.97-fold | P = 0.9745 |
|  | **BT16** | 3.80-fold | P < 0.0001 | 7.49-fold | P < 0.0001 | 1.32-fold | P = 0.0033 |
| **HDAC8** | **SNU.AT/RT-9** | 1.98-fold | P = 0.0001 | 1.67-fold | P = 0.0004 | 1.49-fold | P = 0.0012 |
|  | **SNU.AT/RT-10** | 1.34-fold | P < 0.0001 | 1.19-fold | P = 0.0017 | 0.93-fold | P = 0.0962 |
|  | **BT12** | 1.83-fold | P = 0.0011 | 1.87-fold | P = 0.0010 | 0.93-fold | P = 0.3463 |
|  | **BT16** | 1.90-fold | P < 0.0001 | 2.40-fold | P < 0.0001 | 1.18-fold | P = 0.0198 |
