# Supplementary material for: Combination Treatment of CI-994 With Etoposide Potentiates Anticancer Effects Through a Topoisomerase II-Dependent Mechanism in Atypical Teratoid/Rhabdoid Tumor (AT/RT)
Source: Front Oncol. 2021 Jul 21;11:648023. doi: 10.3389/fonc.2021.648023 (PMC8337050; doi:10.3389/fonc.2021.648023)
Supplement: Supplementary file 5 [file DataSheet_5.docx]

**Supplementary Table S5. Comparison of HDAC enzyme activity**

| **Cell line** | **Control**  **vs**  **Combination** | | **Control**  **vs**  **CI-994** | | **Control**  **vs**  **Etoposide** | |
| --- | --- | --- | --- | --- | --- | --- |
| **SNU.AT/RT-9** | 1.73-fold, | P = 0.0165 | 1.62-fold, | P = 0.0312 | 1.17-fold, | P = 0.656 |
| **SNU.AT/RT-10** | 2.32-fold, | P = 0.0011 | 1.68-fold, | P = 0.0028 | 1.19-fold, | P = 0.0412 |
| **BT12** | 1.96-fold, | P < 0.0001 | 1.87-fold, | P < 0.0001 | 1.26-fold, | P <0.0001 |
| **BT16** | 2.86-fold, | P < 0.0001 | 2.16-fold, | P < 0.0001 | 1.18-fold, | P= 0.0131 |
