# Supplementary material for: Combination Treatment of CI-994 With Etoposide Potentiates Anticancer Effects Through a Topoisomerase II-Dependent Mechanism in Atypical Teratoid/Rhabdoid Tumor (AT/RT)
Source: Front Oncol. 2021 Jul 21;11:648023. doi: 10.3389/fonc.2021.648023 (PMC8337050; doi:10.3389/fonc.2021.648023)
Supplement: Supplementary file 6 [file DataSheet_6.docx]

**Supplementary Table S6. Percentage of cell population in early apoptosis**

| **Cell line** | **Control** | **CI-994** | **Etoposide** | **Combination** |
| --- | --- | --- | --- | --- |
| **SNU.AT/RT-9** | 7.5±6.3 % | 14.8±6.2 % | 22.7±6.7 % | 46.4±2.5 % |
| **SNU.AT/RT-10** | 15.4±4.5 % | 32.0±4.5 % | 26.5±3.2 % | 51.6±3.6 % |
| **BT12** | 2.3±0.2 % | 25.1±1.2 % | 14.5± 4.7% | 46.0±4.0 % |
| **BT16** | 1.7±0.3% | 5.6±0.9 % | 12.1±4.4 % | 38.7±2.0 % |
