# Supplementary figures and images for: Combination Treatment of CI-994 With Etoposide Potentiates Anticancer Effects Through a Topoisomerase II-Dependent Mechanism in Atypical Teratoid/Rhabdoid Tumor (AT/RT)
Source: Front Oncol. 2021 Jul 21;11:648023. doi: 10.3389/fonc.2021.648023 (PMC8337050; doi:10.3389/fonc.2021.648023)

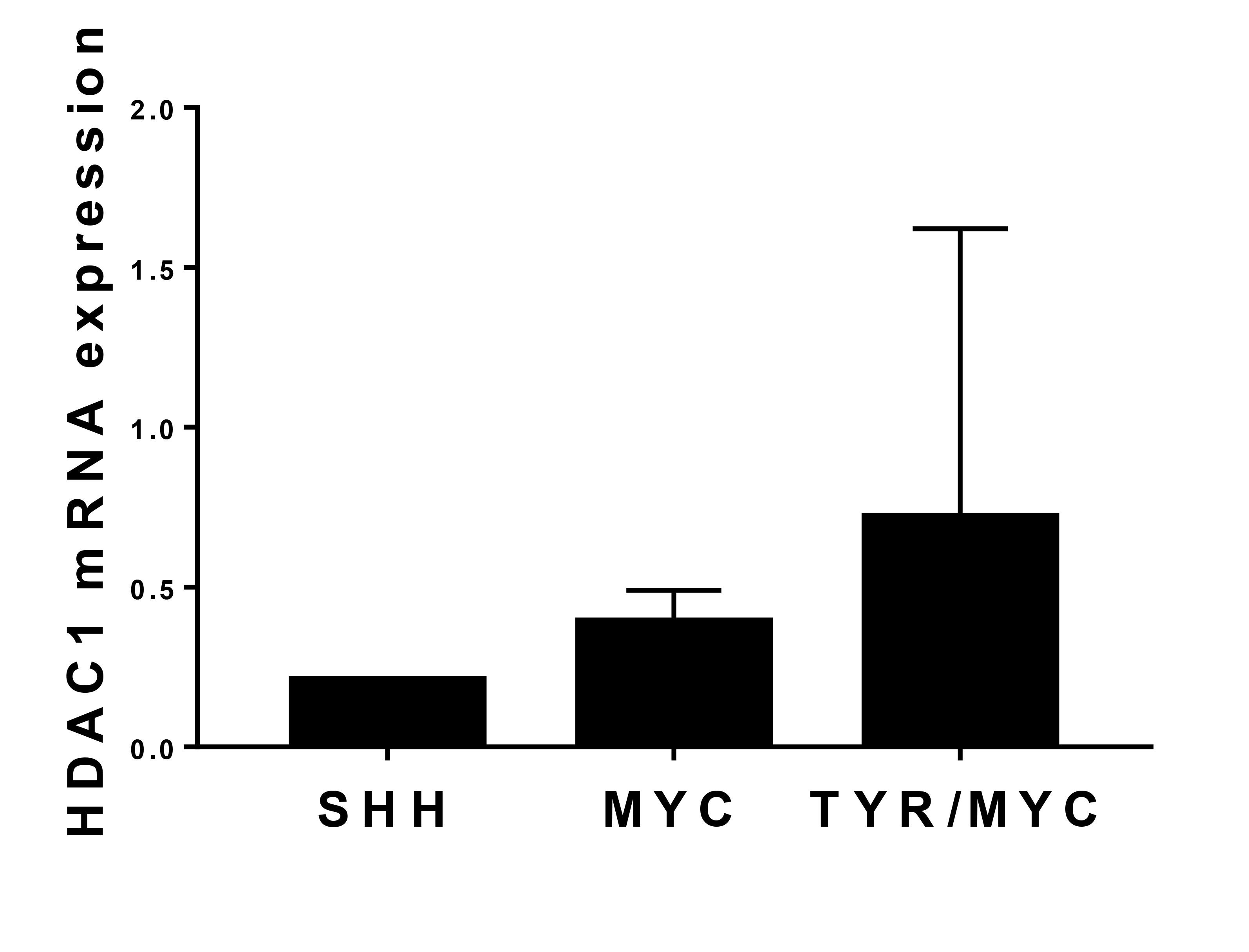

Supplement: Supplementary file 7 [file Image_1.tif]

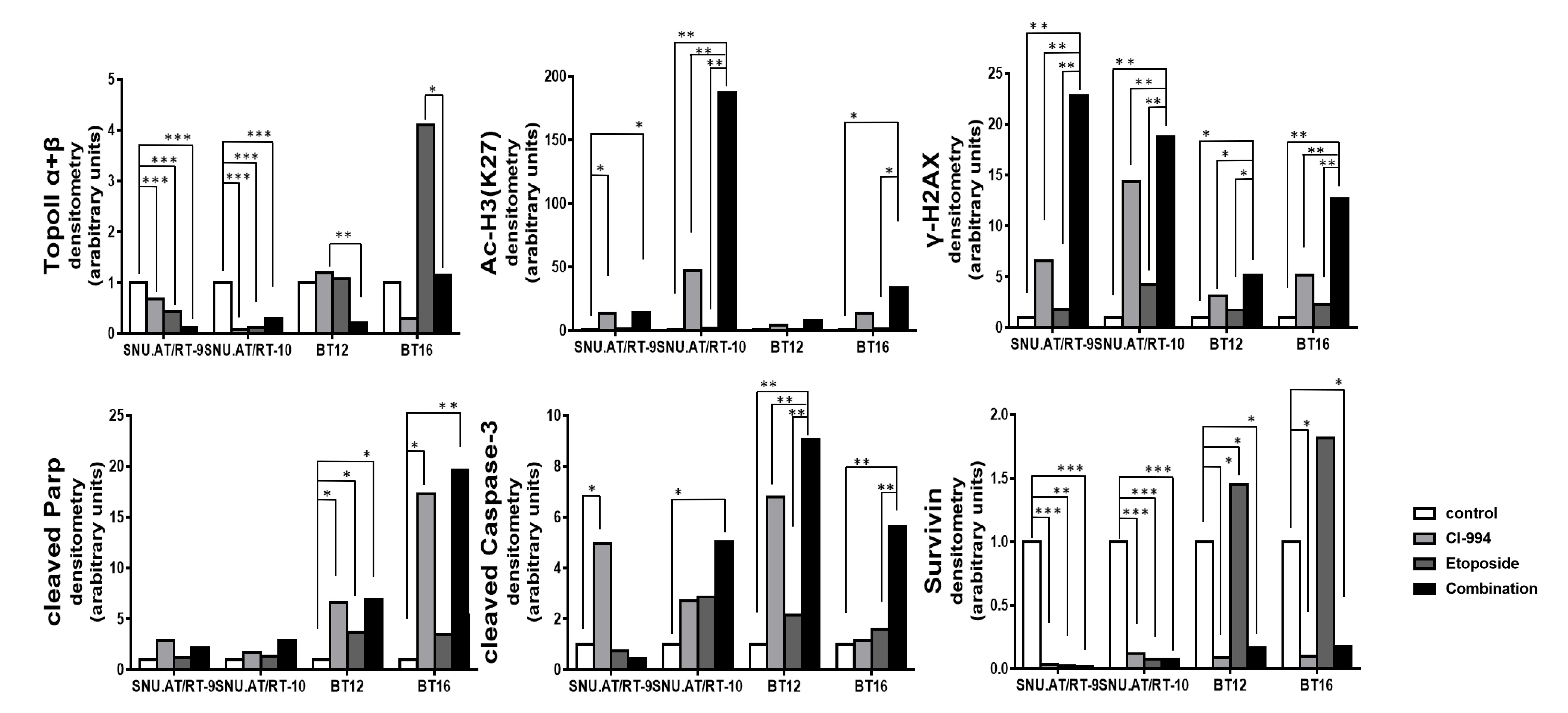

Supplement: Supplementary file 8 [file Image_2.tif]

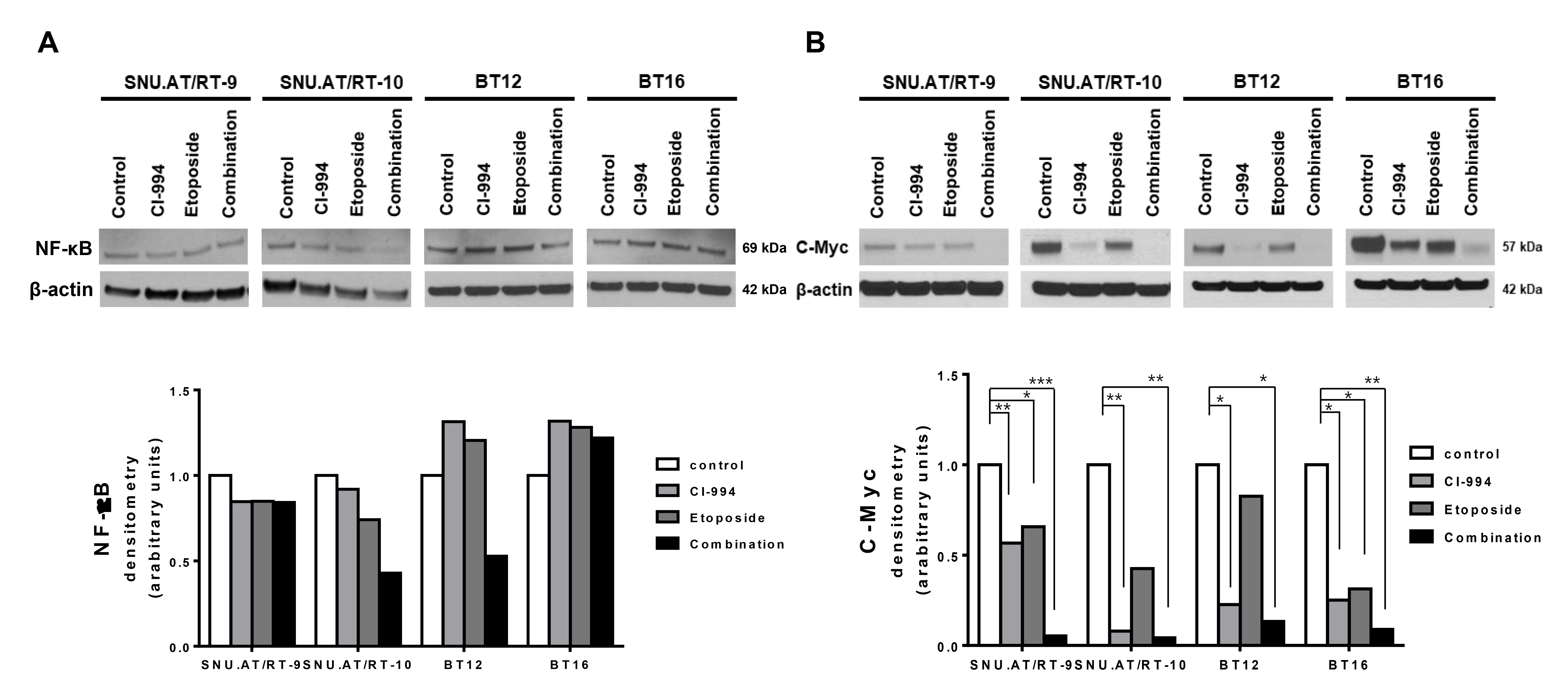

Supplement: Supplementary file 9 [file Image_3.tif]
